# Interstitial deletions of chromosome 1p: novel 1p31.3p22.2 microdeletion in a newborn with craniosynostosis, coloboma and cleft palate, and review of the genomic and phenotypic profiles

**DOI:** 10.1186/s13052-022-01232-7

**Published:** 2022-03-04

**Authors:** Gregorio Serra, Vincenzo Antona, Mario Giuffrè, Ettore Piro, Sergio Salerno, Ingrid Anne Mandy Schierz, Giovanni Corsello

**Affiliations:** Department of Health Promotion, Mother and Child Care, Internal Medicine and Medical Specialties “G. D’Alessandro”, University of Palermo, Palermo, Italy

**Keywords:** 1p31.1 deletion syndrome, Contiguous gene syndrome, Chromosome 1, Array-CGH, Case report

## Abstract

**Background:**

Rearrangements of unstable DNA sequences may alter the structural integrity or the copy number of dose-sensitive genes, resulting in copy number variations. They may lead more frequently to deletions, in addition to duplications and/or inversions, which are the underlying pathogenic mechanism of a group of conditions known as genomic disorders (or also contiguous gene syndromes). Interstitial deletions of the short arm of chromosome 1 are rare, and only about 30 patients have been reported. Their clinical features are variable, in respect of the extent of the deleted region. They include global developmental delay, central nervous system (CNS) malformations, craniosynostosis, dysmorphic face, ocular defects, cleft palate, urinary tract anomalies and hand/foot abnormalities.

**Case presentation:**

Hereby, we report on an Italian female newborn with craniosynostosis, facial dysmorphisms including bilateral microphthalmia and coloboma, cleft palate, and a severe global developmental and growth delay, associated to a 1p31.3p22.2 deletion of 20.7 Mb. This was inherited from the healthy mother, who was carrier of a smaller (2.6 Mb) deletion included within the centromeric region (1p22.3p22.2) of the same rearrangement, in addition to a translocation between chromosomes 1p and 4q. The deleted region of the proband contains about ninety genes. We focus on the genotype–phenotype correlations.

**Conclusions:**

The results of the present study further confirm that microdeletions at 1p31.3 constitute a contiguous gene syndrome. It is hard to establish whether the critical rearrangement of such syndrome may involve the centromeric band p22.3p22.2, or more likely do not, also in light of the genomic profile of the healthy mother of our patient. Neonatologists and pediatricians should take into consideration 1p31 microdeletion in cases of developmental and growth delay associated to craniosynostosis, peculiar facial dysmorphisms, cleft palate and hand/foot abnormalities. The present report provides new data about 1p31 microdeletion syndrome, in view of a better characterization of its genomic and phenotypic profile.

## Background

Rearrangements of unstable DNA sequences may alter the structural integrity or the copy number of dose-sensitive genes, resulting in copy number variations (CNVs). CNVs mainly occur in some genomic regions flanked by highly homologous sequences, defined Segmental Duplications (SDs) or Low Copy Repeats (LCRs), characterized by high similarity, and at greater risk of mismatch (homologous non-allelic recombination, NAHR) [1]. This may cause more frequently deletions, in addition to duplications and/or inversions, which represent the underlying pathogenic mechanism of a group of conditions known as genomic disorders (or also contiguous gene syndromes) [2]. In recent years, the increasing availability and clinical application of high resolution array comparative genomic hybridization (a-CGH) allowed the identification of a growing number of microdeletions and neurogenetic syndromes [2–4]. Interstitial deletions of the short arm of chromosome 1 are rare, with only about 30 patients reported [1]. They present with variable clinical manifestations, in respect of the extent of the deleted region, including global developmental delay, central nervous system (CNS) malformations, dysmorphic features, urinary tract anomalies, as well as craniosynostosis, ocular defects, cleft palate and hand/foot abnormalities [5]. Hereby, we report on an Italian female newborn with craniosynostosis, facial dysmorphisms including bilateral microphthalmia and coloboma, cleft palate and a severe global developmental and growth delay, associated to a 1p31.3-p22.2 deletion. Her clinical and genomic findings were compatible with a 1p31 microdeletion syndrome diagnosis. The 20.7 Mb rearrangement was inherited from the healthy mother, who was carrier of a much smaller (2.6 Mb) deletion included within the centromeric region (1p22.3-p22.2) of the same genomic abnormality of the daughter, in addition to a translocation between chromosomes 1p and 4q. The deleted region of the proband contains about ninety genes, but we focused on those which may play a role in determining her phenotype, in an attempt to suggest possible genotype–phenotype correlations also in light of the maternal genomic profile.

## Case presentation

**Fig. 1:**
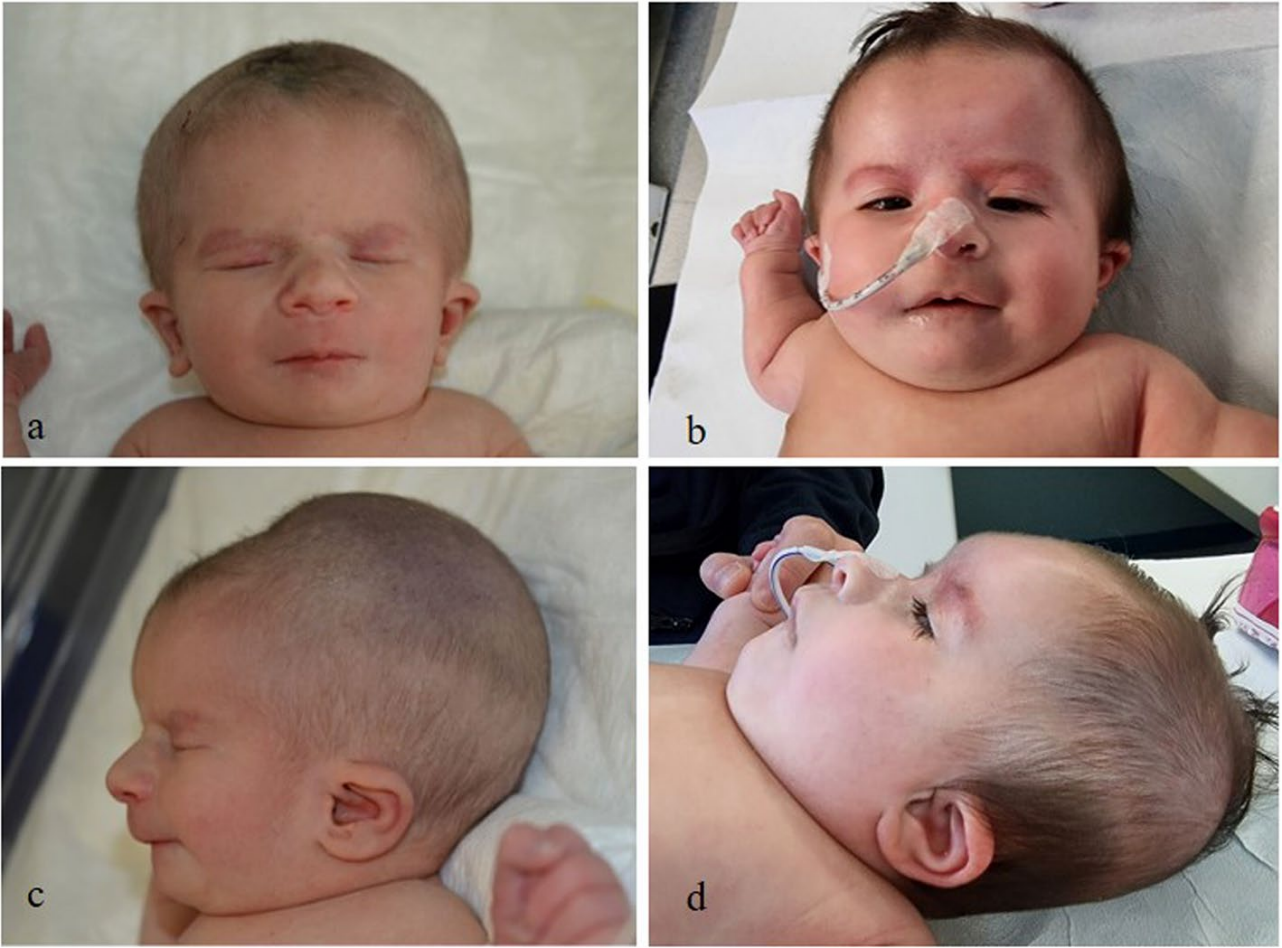
**a** and** b** Patient’s front view at birth and age 4 months: high forehead, frontal bossing, round face, hypotelorism, bilateral microphtalmia, convergent strabismus, epicanthal folds, narrow and down slanting palpebral fissures, broad and depressed nasal root, bulbous tip, anteverted nares, prominent columella, long and hypoplastic philtrum, thin lips with “M” shaped mouth. **c **and** d** Lateral view at birth and age 4 months: brachicephaly with flattened occiput, cupped, small and low-set left ear with thick helix, microretrognathia

**Fig. 2:**
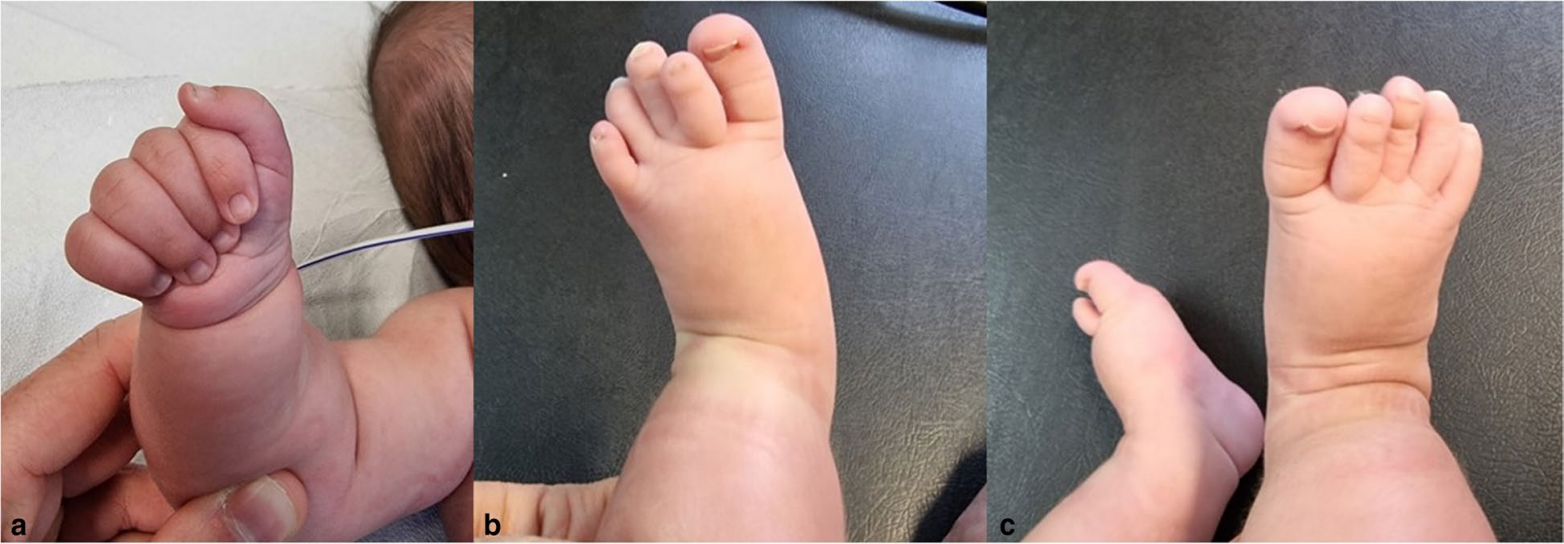
**a** Adducted thumb. **b **and** c** Bilateral calcaneovalgus talipes, crowded toes with nail dysplasia, broad first, proximal position of the second and clinodactyly of the fourth and fifth ones

A female newborn, first child of healthy and nonconsanguineous parents, was born at term by spontaneous delivery. Family history disclosed two relatives, on the maternal side (brothers of maternal grandmother and great-grandfather), affected with cleft palate. Pregnancy was marked by fetal growth restriction, which arose during the third trimester. Apgar scores were 6, 8 and 9, at 1, 5 and 10 min respectively. At birth, anthropometric measures were as follows: weight 2090 g (3^rd^ centile, -1.91 standard deviation, SD), length 44 cm (3^rd^ centile, -1.88 SD) and occipitofrontal circumference (OFC) 33 cm (43^th^ centile, -0.18 SD). Due to difficulties in adapting to extrauterine life requiring non-invasive ventilatory support (continuous positive airway pressure administration), she was transferred to the Neonatal Intensive Care Unit. At admission, physical examination showed high forehead, frontal bossing, brachycephaly with flattened occiput, round face, hypotelorism, bilateral microphthalmia, convergent strabismus, epicanthal folds, narrow and down slanting palpebral fissures, broad and depressed nasal root, bulbous tip, anteverted nares, prominent columella, long and hypoplastic philtrum, thin lips with “M” shaped mouth, cleft palate and microretrognathia. Cupped and small ears (with low-set of the left one) with thick helix, and incisions of the upper gingival mucosa completed her craniofacial profile (Fig. 1a/b/c/d). *Pectus excavatum* and widely spaced nipples were also observed. Bilateral adducted thumbs, *calcaneus*-*valgus* talipes and crowded toes (broad first, proximal position of the second and clinodactyly of the fourth and fifth ones) with nail dysplasia, outlined the abnormalities of hands and foot (Fig. 2a/b/c). A severe generalized hypotonia, in addition to poor reactivity, spontaneous motor activity and suction, as well as impaired swallowing and decreased archaic reflexes, marked her neurological features. After birth she suffered from mild respiratory distress, which needed oxygen support until the 15^th^ day of life. Laboratory analyses including complete blood count, serum electrolytes, liver and kidney function tests, and hormonal levels (thyroid stimulating hormone, TSH; basal growth hormone, GH, performed during the first week of life) showed normal results. Head ultrasound (US) revealed isolated moderate widening of the III ventricle, along with kinked *corpus callosum*. Abdominal US showed hypertrophy of the left hepatic segments, and dysmorphic gallbladder. US heart evaluation showed pulmonary hypertension (pressure gradient around 40–50 mmHg) and patent *foramen ovale*. Ophthalmological evaluation disclosed bilateral coloboma of iris and optic nerve, extending to the posterior pole, arteriovenous shunts of the retinal vessels, as well as bilateral esotropia.

**Fig. 3:**
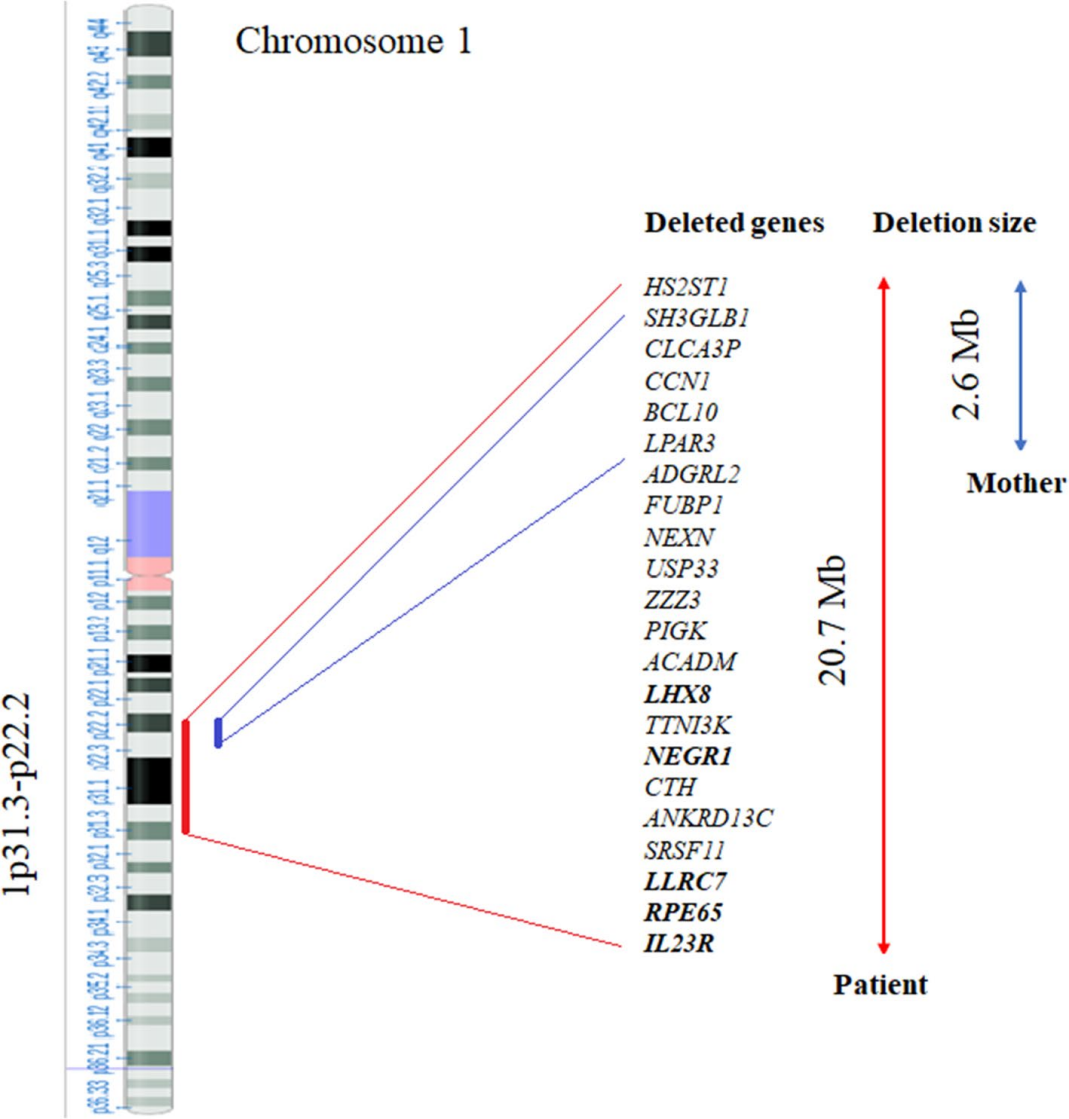
Gene content in relation to the size of deletions between the patient and her mother

aCGH analysis (100–150 Kb resolution, genome assembly GRCh37.p13) identified a 1p31.3-p22.2 deletion of 20.7 Mb and indicated the positions 67,721,572 and 88,415,438 as the breakpoints of the rearrangement. The deleted region involved about ninety genes, including *IL23R*, *RPE65, LRRC7, SRSF11, ANKRD13C, CTH, NEGR1, TTNI3K, LHX8, ACADM, PIGK, ZZZ3, USP33, NEXN, FUBP1, ADGRL2, LPAR3, BCL10, CCN1, CLCA3P,* and *SH3GLB1*, which are mainly those with higher scores of probability of loss-of-function (LoF) intolerance (pLI) (Fig. 3). Fluoresence in situ hydridization (FISH) was then performed in both parents, showing normal results in the father and a translocation between the short arm of chromosome 1 and the subtelomeric region of the long arm of chromosome 4, t(4;1)(q35;p31.1p31.1), in the healthy mother, identifying thus the cause of the genetic abnormality of the proband. Moreover, the aCGH analysis performed in the latter (65 Kb resolution, genome assembly GRCh37.p13) showed a 1p22.3-p22.2 deletion of 2.6 Mb, enclosed within the same rearrangement of the daughter and with overlapping centromeric breakpoint (from positions 85,869,876 to 88,477,895) (Fig. 3).

The absence of some specific signs (syndactyly of hands and/or feet, ocular proptosis, ankylosis/synostosis of limb bones and cervical vertebrae), and the type of craniosynostosis (brachycephaly instead of acrocephaly for early fusion of coronal and sagittal sutures) made the present patient’s clinical picture poorly compatible with the main syndromic craniosynostoses, like Apert, Crouzon and Pfeiffer syndromes. Among them, Pfeiffer syndrome (PS) may have the highest phenotypic overlap with our newborn, owing to craniosynostosis and wide first toes (present also in our patient). However, the absence of enlarged and medially deviated thumbs (which were conversely adducted), midfacial hypoplasia and ocular proptosis (which are all distinctive and prevalent features of PS) in our patient, who instead presented bilateral microphthalmia and coloboma, did not direct the diagnostic suspicion towards PS. Furthermore, the absence of macrocephaly and brachydactyly made the diagnostic hypothesis not very consistent also with Muenke syndrome. Then, based both on the phenotype and the genetic findings, it was not considered necessary to proceed with target next generation sequencing (NGS) analysis of craniosynostosis-related genes (*FGFR1*, *FGFR2*, *FGFR3*, *TWIST*, *EFNB1* and *TCF12*).

The following clinical evolution was characterized by persistent severe generalized hypotonia, developmental delay and feeding difficulties, which needed enteral nutrition by nasogastric tube during the whole hospital stay. She was discharged at 2 months and 20 days of age in good general conditions, despite poor weight and length growth, and included in a multidisciplinary follow-up. Hearing screening through transient-evoked otoacoustic emissions (TEOAEs) revealed abnormal results. In order to ascertain and characterize the hearing loss, an audiological assessment was started. It included serial auditory brainstem response (ABR) evaluations at 2, 4 and 6 months of age, which disclosed, and then confirmed, a bilateral moderate sensorineural hypoacusis requiring hearing-aid, applied at 6 months. Moreover, at the age of 4 months, a three-dimensional reconstruction computed tomography (3D-CT) of the cranium showed bilateral coronal craniosynostyosis, due to reduced representation for fusion of both such sutures. No further alterations of the other sutures were observed, while reduction of the antero-posterior diameter (APD, 106 mm measured on a plan passing through the lateral ventricles), and increase of the transversal one (TD, 114 mm measured on a plan passing through the two temporal bones), with Cephalic Index (CI = TD/APD × 100) 107, compatible with severe brachycephaly (normal CI 75–90, mild alteration 91–93, moderate 94–97, severe > 97; brachycephaly if CI > 90%, dolichocephaly if CI < 76%) [6], were documented. Furthermore, head US ruled out hydrocephalus. She currently is 1 year and 26 days old, and her anthropometric measures, according to World Health Organization growth chart for neonatal and infant close monitoring [7], are: weight 6650 g (< 1^st^ centile, -2.67 SD), length 65 cm (< 1^st^ centile, -2.68 SD) and OFC 43.2 cm (8^th^ centile, -1.42 SD). She has a severe global developmental delay, with a central type axial and lower limbs hypotonia, and absent lateral parachute reaction on both sides. She cannot sit unsupported, and is not able to speak two syllables. She can turn the head, follow and reach an object (red cube and suspended red ring) in the midline with both her hands. Blood examination, heart and abdominal US show no further abnormalities.

## Discussion and conclusions

Although chromosome 1 is the largest chromosome in the human genome, only about 30 patients with interstitial microdeletions of its short arm were reported [1, 8–10]. Few of them [11–13] were described in the current microarray era and, then, the centromeric and telomeric breakpoints delimiting the exact rearranged region and the precise number of genes involved in the microdeletions were not clearly defined. Clinical features of these subjects include developmental delay, seizures, CNS malformations, macrocephaly (40% of cases), elongated or rounded face with prominent nose, micro/retrognathia, half-opened mouth, short neck, congenital heart malformations, hernia, hand/foot malformations, renal anomalies, abnormal external genitalia, joint hyperlaxity and *cutis laxa* [1, 5]. We identified a 20.7 Mb deletion at chromosome 1p31.3-p22.2, in an Italian female newborn with craniosynostosis (brachycephaly for premature fusion of both coronal sutures), bilateral microphthalmia and coloboma in addition to other facial dysmorphic features, cleft secondary palate, hands and foot abnormalities and severe developmental and growth delay. Unlike to the few reported cases, our patient harbors a 1p31 microdeletion spanning towards the centromere to reach the p22.2 band (less frequently reported than the involvement of the telomeric region p32) [13], and did not show either renal malformations or hormonal (TSH and GH) defects (Table 1).

**Table 1:** Comparison of present patient phenotype with that of interstitial deletions of chromosome 1p

	**Respiratory**	**Cardiovascular**	**Ear, nose, throat**	**Gastrointestinal**	**Infectious diseases**	**Endocrinology**	**Nephrology**	**Ophthalmology**	**Neurology**	**Musculoskeletal, skin**	**Genital anomalies**	**Genetics**	**Methods**
**Present patient**	Respiratory distress	Pulmonary hypertension, patent *foramen ovale*	Broad and depressed nasal root, bulbous tip, anteverted nares, prominent columella, long and hypoplastic philtrum; thin lips with “M” shaped mouth, cleft palate, incisions of the upper gingival mucosa, microretrognathia; cupped and small ears (with low-set of the left one) with thick helix, bilateral moderate sensorineural hypoacusis	Hypertrophy of the left hepatic segments, dysmorphic gallbladder	No infection diagnosed	No hormonal defects	No urinary tract abnormalities	Hypotelorism, bilateral microphthalmia, convergent strabismus, epicanthal folds, narrow and down slanting palpebral fissures, bilateral coloboma of iris and optic nerve, arteriovenous shunts of the retinal vessels	Severe global developmental delay, isolated moderate widening of the III ventricle, kinked *corpus callosum*	High forehead, frontal bossing, brachycephaly with flattened occiput, round face, *pectus excavatum*, widely spaced nipples, bilateral adducted thumbs, talipes calcaneovalgus and crowded toes (broad first, proximal position of the second and clinodactyly of the fourth and fifth ones) with nail dysplasia	No abnormalities	1p31.3-p22.2 deletion of 20.7 Mb (from 67,721,572 to 88,415,438), inherited from the healthy mother, carrier of a smaller (2.6 Mb) deletion within the same rearrangement of the daughter (1p22.3p22.2) and with overlapping centromeric breakpoint (from 85,869,876 to 88,477,895), in addition to a translocation t(4;1)(q35;p31.1p31.1)	aCGH
**Yieh et al., 2019 **[13] **Twin A**	Abnormal lobation of lungs (right lung with 1 fissure, left lung with no fissures)	Cardiomegaly, 2 midmuscular ventricular septal defects, high *ostium secundum* atrial septal defect, patent *ductus arteriosus*	Macroglossia, cleft palate, microretrognathia, low set ears, anteverted nostrils	Meconium plugs, surgical necrotizing enterocolitis, hypoplastic spleen No congenital heart malformations	No infection diagnosed	Hypoplastic adrenal glands, presumed cortisol deficiency	Bilateral simple cysts, salt‐wasting nephropathy, hypercalciuria, hematuria	Right eye optic nerve and retinal coloboma	*Corpus callosum* dysgenesis, ventriculomegaly, cortical or subcortical calcifications within the right frontal and parietal lobes, possible tethered spinal cord	Abnormal skeletal proportions with smaller than expected crown‐rump and crown‐heel length, small hand length, asymmetric clefts within the right aspects of the S3 and S4 vertebral bodies, widely spaced nipples, bifid left hand 5^th^ digit	No abnormalities	1p22.2-p32.2 deletion of 31.67 Mb	SNP-microarray
**Yieh et al., 2019 **[13] **Twin B**	Chronic lung disease, concern for airway malformation	Multiple small midanterior muscular ventricular septal defects, patent *ductus arteriosus*, pulmonary hypertension, *ostium* *secundum* ASD	Macroglossia, retroflexed epiglottis, retrodisplaced base of tongue, cleft of hard and soft palate, retrognathia, low set ears	Suspected Hirschsprung's disease, surgical necrotizing enterocolitis	*Enterobacter* pyelonephritis, MDR *E. coli* pyelonephritis	Low vitamin D, hypocalcemia, normal ACTH stimulation test	Bilateral simple cysts, salt‐wasting nephropathy, nephrocalcinosis, hematuria	Pale, hypoplastic optic nerves	Macrocephaly, ventriculomegaly, agenesis of the *corpus* *callosum*, subcortical punctate calcifications, diffusely abnormal gyral pattern, hypoplastic chiasm, tethered spinal cord, bilateral grade 3/4 IVH	Widely spaced nipples, shortened humeri, triphalangeal right 1^st^ digit	No abnormalities	1p22.2-p32.2 deletion of 31.66 Mb	SNP-microarray
**Rivera-Pedroza et al., 2017 **[5]	No respiratory problems	No congenital heart malformations	Cleft palate, low-set ears, micrognathia	No gastrointestinal anomalies	Respiratory tract infection	No hormonal defects	Bilateral hypoplasia with abnormal cortical echogenicity and altered corticomedullary differentiation	Hypotelorism and severe exophthalmos, absence of eyelids, ectopia lentis, sclerocornea	Biventricular enlargement, collapse of the third and fourth ventricles, small posterior fossa, focal intracerebral hemorrhage in the left temporal lobe, obstructive hydrocephalus with enlargement of temporal regions	Prominent midfrontal line, cloverleaf skull, *cutis laxa*	No abnormalities	1p31.1-p31.3 deletion of 18.6 Mb (63,871,758– 82,484,133)	SNP-microarray and MLPA
**Thakur et al., 2017 **[23]	Respiratory Distress	Patent *foramen ovale*	Flat nasal bridge with anteverted nostrils; small, low-set, posteriorly rotated ears with overfolded helices; long philtrum, micrognathia, high arched palate Severe bilateral sensorineural hearing loss	No gastrointestinal anomalies	No infection diagnosed	Hypopituitarism	No abnormalities	Hypotelorism, almond-shaped eyes, infraorbital creases	Absence of the *septum pellucidum*, mild lobularity of the medial cortical surfaces, ventricular enlargement, several periventricular cystic areas, diminutive sella, ectopic posterior pituitary gland, nonvisualization of the anterior pituitary gland, hypoplastic *corpus callosum*, global developmental delay	Trigonocephaly with a prominent brow and metopic ridge; widely spaced, hypoplastic nipples, clenched hands with deep palmar creases and long-appearing fingers; fifth digit clinodactyly of both feet, sacral dimple	Small scrotum and phallus; unilateral cryptorchidism	1p31.1-p31.3 deletion of 8.04 Mb	SNP-microarray
**Labonne et al., 2016 **[21]	No respiratory problems	No congenital heart malformations	Macrocephaly, prominent forehead, frontal bossing, low-set ears, narrow nose and thin lips	No gastrointestinal anomalies	No infection diagnosed	No hormonal defects	No abnormalities	No anomalies	Developmental delay, intellectual disability, subarachnoid intraventricular hemorrhage with layering in the posterior fossa, right anterior communicating artery aneurysm, ADHD	No anomalies	No abnormalities	1p31.3-p32.2 deletion of 9.45 Mb (57,633,718- 67,087,056)	microarray

*aCGH *array comparative genomic hybridization, *ADHD *attention deficit hyperactivity disorder, *ASD *atrial septal defect, *FISH *fluorescence in situ hybridization, *IVH *intraventricular hemorrhage, *MDR *multidrug resistant, *MLPA* multiplex ligation-dependent probe amplification, *SNP *single nucleotide polymorphism

Indeed, the interstitial deletions previously described were mainly smaller than ours and involved the chromosomal region towards the telomere including some genes which are spared in present newborn. Among these patients, the one described by Rivera-Pedroza et al.[5] is more similar from a genetic as well as clinical point of view. This female newborn carried a 1p31.1p31.3 deletion of 18.6 Mb, and presented with a phenotype considerably overlapping with that of our proband, and including cloverleaf skull, round face, hypotelorism, severe exophthalmos with the absence of eyelids, ectopia lentis, sclerocornea, prominent nose, cleft palate, half-opened mouth, microretrognathia, low-set ears, *cutis laxa*, hand/foot abnormalities, congenital heart disease and developmental delay. Compared to our newborn, she additionally had bilateral renal hypoplasia, abnormal external genitalia, hernia, as well as seizures, obstructive hydrocephalus, small posterior cranial fossa and intracerebral hemorrhage.

The deleted region of the present newborn contains about ninety OMIM genes. We paid attention to about 20 of them, which are mainly those considered LoF intolerant according to their haploinsufficiency score (pLI score): *IL23R*, *RPE65, LRRC7, SRSF11, ANKRD13C, CTH, NEGR1*, *TTNI3K, LHX8, ACADM, PIGK, ZZZ3, USP33, NEXN, FUBP1, ADGRL2, LPAR3, BCL10, CCN1, CLCA3P,* and *SH3GLB1* (Fig. 3).

Among these, *IL23R* (interleukin 23 receptor) may have a potential association with craniosynostosis, as already suggested by previous genetic and population analyses [5]. Thus, although partially deleted, *IL23R* may be related to the craniosynostosis also in our patient. *LHX8* (LIM homeobox 8) encodes a transcriptional regulator of the family LIM-homeobox, which is expressed in the first branchial arch and the basal forebrain. Its haploinsufficiency was found in patients with cleft palate in addition to microcephaly and severe learning difficulties, carrying smaller chromosome 1 deletions, and it is deleted also in our *proposita*. Moreover, this gene is highly expressed in connective tissue, skin and its appendages (tooth formation). Therefore, it is hard to establish a clear relation between its defect and the cutaneous abnormalities observed in 1p31 microdeletion patients, as well as the nail dysplasia observed in our newborn. *RPE65* (retinoid isomerohydrolase RPE65) encodes for a protein which is a component of the vitamin A visual cycle of the retina. It is a member of the carotenoid cleavage oxygenase superfamily, and performs the essential enzymatic isomerization step in the synthesis of 11-cis retinal. Its mutations are associated with early-onset severe blinding disorders such as Leber congenital amaurosis, and it may be linked to the ocular abnormalities (microphthalmia, coloboma, retinal vessels malformations) present in our patient. *LRRC7* (leucine rich repeat containing 7), *GADD45A* (growth arrest and DNA damage inducible alpha), and *NEGR1* (neuronal growth regulator 1) are candidate genes for the developmental delay, and the psychiatric and language disorders reported in 1p31 microdeletion patients [11, 14, 15], and two of them (*LRRC7* and *NEGR1*) are deleted in the proband (who actually presents with a severe neuromotor and language delay). Conversely, *NFIA* (nuclear factor IA) gene, which was associated to CNS malformations (*corpus callosum* and cerebellar anomalies, ventriculomegaly, hydrocephalus, tethered spinal cord, type I Chiari malformation) craniofacial abnormalities (metopic synostosis, facial dysmorphisms), developmental delay and genitourinary tract defects [16–22], is spared in the *proposita*, who actually shows only minimal morphological CNS abnormalities (isolated moderate widening of the III ventricle and kinked *corpus callosum*) with no genitourinary abnormalities. These findings agree with the possible involvement of such gene in renal development, but it seems unlikely its role in causing the CNS defects of present patient, although indirect mechanisms (i.e. disruption of regulatory elements) may not be excluded. *LEPR* (leptin receptor) and *JAK1* (Janus kinase 1) haploinsufficiency were associated to abnormal pituitary development and obesity [23]. Also these genes are not included in the deletion of our newborn, who does not show to date either hormonal (TSH and GH) abnormalities or other clinical signs of endocrine dysfunction, including excess weight.

Then, we identified and analyzed, according to literature data, the genes which may be responsible for the clinical findings of our newborn. It is hard to establish whether the other genes in the deleted centromeric region p22.3-p22.2 (i.e., *LPAR3*, *BCL10*, *CCN1*, *SH3GLB1, HS2ST1*) may also play a causative role for the phenotype of present patient, and be then considered within the critical rearrangement of 1p31 microdeletion syndrome, or more likely do not, also in light of the genomic profile (deletion of such p22.3-p22.2 genes) of the healthy mother of the proband (Fig. 3). The results of present study further confirm that microdeletions at 1p31.3 constitute a contiguous gene syndrome, whose genotype–phenotype correlations are still only partially elucidated, due to variability both in phenotype expressivity and deletion size typical of genomic disorders [24–26]. Further characterization of this genomic region, analysis of other patients, and functional studies will provide more insights both on the number and type of critical genes and their impact on this contiguous gene syndrome.

1p31 microdeletion syndrome must be distinguished from *FGFR3*-related craniosynostoses, from which it differs for associated manifestations and underlying pathogenic (genetic and molecular) mechanisms. Specifically, some suggestive clinical signs (syndactyly of hands and/or feet, ocular proptosis, ankylosis/synostosis of limb bones and cervical vertebrae), and the type of craniosynostosis (acrocephaly for early fusion of coronal and sagittal sutures, rather than brachicephaly) may help in the differential diagnosis, since they are more frequently observed in the main syndromic craniosynostoses, like Apert, Crouzon, and Pfeiffer syndromes. Neonatologists and pediatricians should take into consideration interstitial deletions of chromosome 1 in cases of developmental and growth delay associated to craniosynostosis, peculiar facial dysmorphisms, cleft palate and hand/foot abnormalities [27]. The present report provides new data about 1p31 microdeletion syndrome, in view of a better characterization of its genomic and phenotypic profile.

## Data Availability

The datasets used and analyzed during the current study are available from the corresponding author on reasonable request.

## Abbreviations

3D-CT: Three-dimensional reconstruction computed tomography
a-CGH: Array comparative genomic hybridization
ABR: Auditory brainstem response
APD: Antero-posterior diameter
CI: Cephalic index
CNS: Central nervous system
CNVs: Copy number variations
FISH: Fluoresence in situ hydridization
GH: Growth hormone
LoF: Loss of function
OFC: Occipitofrontal circumference
OMIM: Online Mendelian Inheritance in Man
pLI: Probability of loss-of-function intolerance
PS: Pfeiffer syndrome
SD: Standard deviation
TD: Transversal diameter
TEOAE: Transient-evoked otoacoustic emissions
TSH: Thyroid stimulating hormone
US: Ultrasonography
